# Radiofrequency Cautery: A Safe Option for Patients With Indwelling Devices

**DOI:** 10.7759/cureus.95809

**Published:** 2025-10-31

**Authors:** Andrew M Alfred, Aaron M Kessler, Dena Danji, Arpan Kohli

**Affiliations:** 1 Anesthesiology, West Virginia University School of Medicine, Morgantown, USA

**Keywords:** dbs, deep brain stimulators (dbs), lead impedance, non-cardiac implantable electrical devices, perioperative management, radiofrequency cautery, scs, spinal cord stimulators (scs)

## Abstract

The increasing prevalence of non-cardiac implantable electrical devices (NCIEDs), including deep brain stimulators (DBS), spinal cord stimulators (SCS), and an increasing list of other devices, presents a significant challenge to conscientious perioperative care teams. The major anticipated operative complications of having a pre-existing indwelling device are twofold. For the first category, there is the profound threat of direct thermal injury to tissue surrounding the device in question, as well as the lateral thermal spread. Of course, when the surrounding tissue is neural and may involve deep brain structures or spinal cord structures, these concerns are all the more pressing. As for the second category of anticipated danger, there is the concern of destructive interaction between electrocautery and the device itself. Considering the total investment made for the patient to acquire an implantable device and follow-up for adjustment and tuning, as well as the potential for uncontrolled or unintentional delivery of electricity, this is a significant concern. Such interactions could conceivably lead to worsened control of pre-existing neurologic conditions or chronic pain, making a recovery to baseline unpredictable or even impossible without further post-operative intervention. We present the case of a 56-year-old male patient with diabetic neuropathy treated with SCS and Parkinson’s disease treated with DBS undergoing triple-vessel coronary artery bypass grafting (CABG). Due to concerns of potential interaction between the cautery device and the NCIEDs, causing direct thermal injury as well as device malfunction, both indwelling devices were powered down pre-operatively. Furthermore, although bipolar electrocautery is a relatively safe and more widely known option for patients with NCIEDs, the caveat of poor hemostasis was presented by the surgical team. With this in mind, the decision was made to proceed with PlasmaBlade (Medtronic, Minneapolis, MN) monopolar radiofrequency cautery, which according to its manufacturer, is notably less likely to cause damage to NCIEDs. The patient tolerated cardiac surgery with no bleeding complications or unintended consequence to the implanted devices, nor was there any apparent or suspected thermal injury to the tissue surrounding the devices.

## Introduction

The presence of indwelling medical devices is an increasingly prevalent concern for peri- and intra-operative care teams. While the perioperative management of implanted cardiac devices such as pacemakers and automated implantable cardioverter-defibrillators is generally well known to practitioners of anesthesiology, the proper perioperative management of non-cardiac implantable electrical devices (NCIEDs) has gained significant importance over recent years, with some in the field calling for the creation of NCIED guidelines similar to those for cardiac devices [1,2]. The list of NCIEDs is ever-expanding to include new peripheral, neuraxial, and intracranial options for management of pain and other neurologic conditions, including Parkinson’s disease, peripheral neuropathy, ischemic pain, phantom limb pain, complex regional pain syndrome, failed back surgery syndrome or persistent spinal pain syndrome type 2, urinary incontinence, obstructive sleep apnea, and more [3]. In addition to spinal cord stimulators (SCS) and deep brain stimulators (DBS), the list of non-cardiac devices has grown to include vagal nerve stimulators, gastric stimulators, phrenic nerve stimulators, bone stimulators, and more, and the use of such devices is initiated increasingly early in treatment algorithms [4,5]. What was once a last resort has become an increasingly common solution. When a patient with such a device, or multiple such devices as in our case, presents for surgery, anticipatory management of potential interactions with surgical equipment is of utmost importance. While perioperative device management is generally characterized by device manufacturers to involve dialing the amplitude of the implanted NCIEDs down to a minimum, placing them in a safe mode, or turning the devices off altogether as well as minimizing the risks of monopolar cautery by selecting appropriate settings and being thoughtful in positioning grounding pads as far as possible from indwelling devices, the risks and potential complications must be characterized on a patient-to-patient basis and managed appropriately [5]. This report describes the case of a patient with both an intracranial and a neuraxial NCIED who presented for open cardiac surgery, demonstrating the importance of proper perioperative management of such devices and elucidating the less discussed option of radiofrequency cautery as a safe and effective surgical tool in this patient population.

## Case presentation

**Figure 1:**
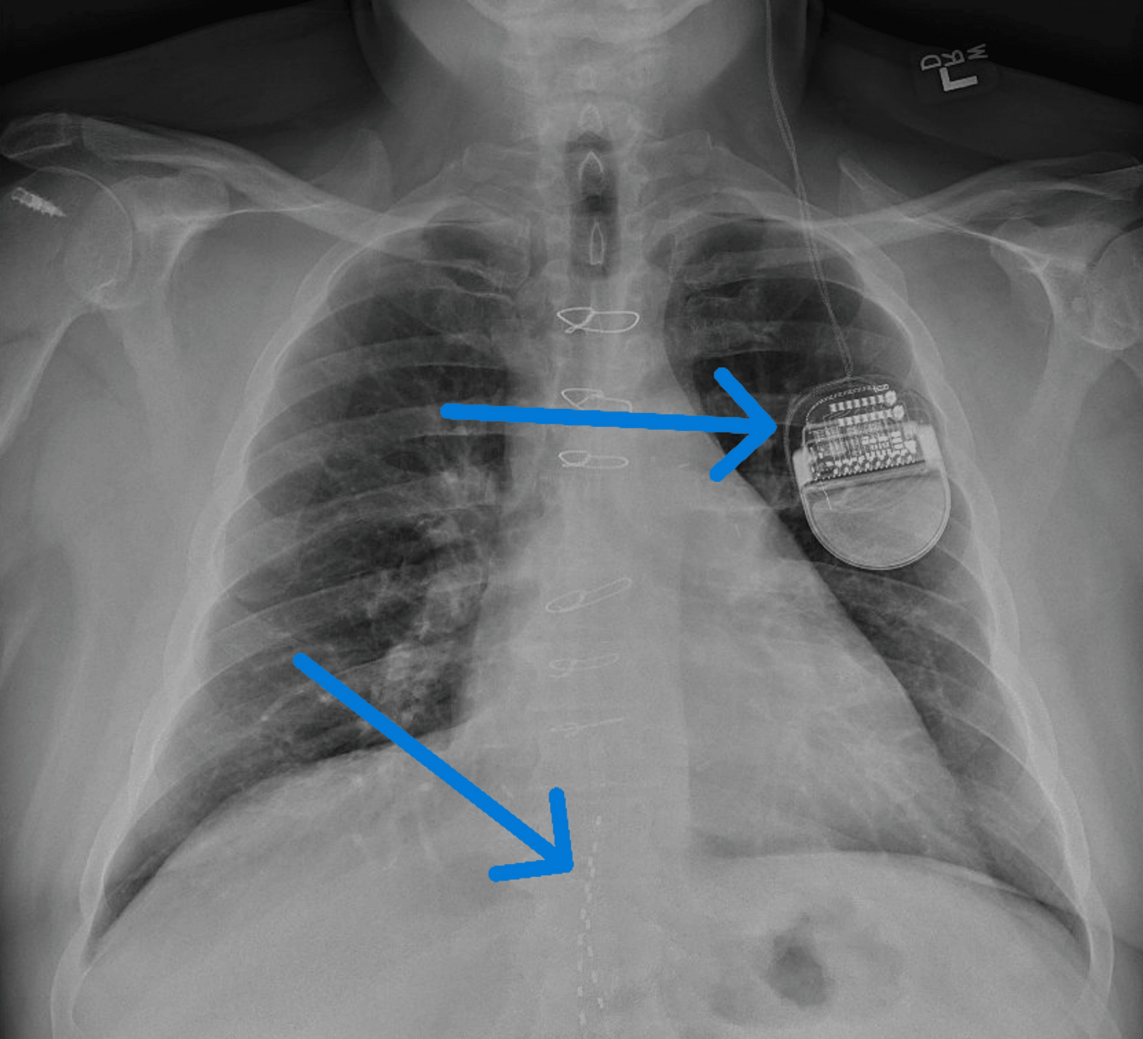
Location of the patient's DBS generator on left chest (upper arrow), SCS leads (lower arrow) also visible inferiorly in the thoracic spine DBS: brain stimulator; SCS: spinal cord stimulator.

**Figure 2:**
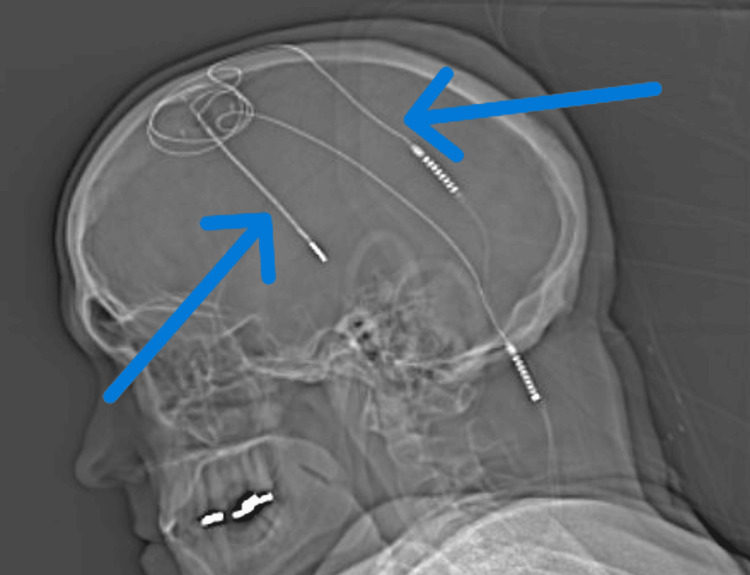
Location of the patient's DBS leads (arrows) DBS: Deep brain stimulator.

A 56-year-old male patient with a known history of diabetes with associated diabetic neuropathy with recently placed SCS (Figure 1), Parkinson’s disease with indwelling DBS (Figures 1, 2), hypertension, hyperlipidemia, and newly diagnosed coronary artery disease presented for triple-vessel coronary artery bypass grafting (CABG) under general anesthesia.

The patient was planned for endoscopic radial artery harvesting as a part of his surgical procedure. Careful management of his NCIEDs was undertaken by the anesthesia team. Both NCIEDs were appropriately powered down using the patient’s own remote, and the request was made for monopolar electrocautery to be avoided. At this point, it became apparent that the endoscopic radial artery harvesting technique was only compatible with monopolar electrocautery.

Instead of proceeding with this surgical technique, discussion between care teams led to the joint decision to proceed with open saphenous vein harvesting instead of the endoscopic radial technique. The open approach allowed the surgical team to utilize a PlasmaBlade (Medtronic, Minneapolis, MN) radiofrequency cautery device, for which the grounding pad was appropriately placed on the hip, contralateral to the SCS generator (i.e. as far from all parts of the patient’s implanted devices as possible). One major concern with this decision was the potential for unpredictable and possibly inadequate hemostasis with the novel cautery device.

Ultimately, no such concerns came to fruition, and the device provided adequate hemostasis as evidenced by the proceduralist's qualitative assessment as well as the lack of transfusion requirement. With the above measures, unwanted interaction between cautery and the devices, including concerns of both device malfunction and thermal injury, was prevented. Following surgery, the patient’s devices were powered back on using his remote in the post-anesthesia care unit. There were no immediate complications related to either of his NCIEDs, and a return to the patient's preoperative baseline progressed as expected.

## Discussion

This case demonstrates the appropriate anticipatory management of a patient with multiple NCIEDs undergoing cardiac surgery. Patients with NCIEDs are at significant risk of device malfunction or thermal injury related to electrocautery when appropriate considerations for their indwelling devices are overlooked. Other significantly concerning interactions would be those between NCIEDs and other sources of electrical current, such as neuromonitoring techniques, defibrillation, cardioversion, electroconvulsive therapy electrodes, and MRI [6].

For our patient, the major concern out of these potential sources was the previously planned use of monopolar electrocautery with the potential for damage to integral neurologic structures. Several alternatives to monopolar electrocautery exist, including bipolar electrocautery, diathermy, ultrasonic scalpel, Harmonic (Ethicon, Cincinnati, OH), and monopolar radiofrequency cautery (e.g., PlasmaBlade), each of which has a unique risk profile for patients with NCIEDs, with some being safer than others [7]. Bipolar electrocautery induces a localized current that exists only between the two electrodes of the cutting device itself. Its ability to provide adequate dissection and hemostasis compared to traditional methods can be subpar. Diathermy refers to the generation of heat via electromagnetic currents and is broadly unsafe in patients with NCIEDs. Ultrasonic scalpel or Harmonic devices transfer heat to tissue precisely and without inducing an electrical current through the patient's body. The use of these devices in patients with cardiac implanted electrical devices has shown promise [8,9].

PlasmaBlade is a form of monopolar radiofrequency cautery, which induces less thermal spread in the affected tissue and uses less energy than traditional forms of cautery. It has been shown to be feasible in procedures for patients with cardiac implantable electrical devices [10]. Manufacturer-specific NCIED-related safety recommendations should be consulted prior to choosing an alternative cautery tool as some manufacturers explicitly disclose the safety of one form of cautery over another. After considering device availability, surgical preference, hemostasis concerns, and NCIED manufacturer recommendations, PlasmaBlade was chosen for this case.

Although PlasmaBlade is technically a monopolar device, it differs from monopolar electrocautery in that it uses radiofrequency to separate and coagulate tissue at a lower temperature than traditional monopolar cautery and in a fashion that reduces the amount of thermal damage to the surrounding tissue [11]. The grounding pad should still be placed far from all portions of all indwelling devices. Despite this, adequate hemostasis was still attainable, as evidenced by the lack of unexpected bleeding and transfusion in our case. This supports the device's use for patients who are having NCIEDs placed or revised, as well as for patients undergoing surgery unrelated to their NCIEDs [12,13].

Beyond awareness of the presence of NCIEDs and seeking alternative cautery, imaging, and neuromonitoring modalities, it is also of significant importance for the perioperative care team to be aware of the location of the implanted device(s), including both the generator and the path of the electrodes. This can assist in assessing a patient’s risk profile if an electrical current were to be induced and can also assist in positioning grounding pads appropriately to decrease that risk maximally. For the patient described herein, the risk of induction in the electrodes would have caused damage to the structures in the vicinity of the epidural space, including the spinal cord as well as to deep brain regions. The risk profile of induction of current in the NCIEDs of such a patient cannot be overstated. While NCIED electrodes present the risk of damage to surrounding tissues, often including vital neurologic structures, the location of the generator and battery must also be considered. Interference with the generator can lead to device reprogramming, malfunction, delivery of inappropriate shock via the electrodes, or damage to tissue immediately surrounding the generator. Perioperative care teams should be aware of common generator locations such as the upper chest, abdomen, and upper buttocks so that generators can be located and disruption of their function avoided. These sites are often recommended by manufacturers due to an appropriate amount of soft tissue within which to comfortably embed the generator as well as patient accessibility. For patients with multiple NCIEDs, the ideal placement is distant from one another to minimize the potential for interference [14,15].

There exist circumstances under which lower risk alternatives to necessary surgical, medical, and imaging tools may not be feasible or available despite suspected risk of deleterious interactions with indwelling devices. In such cases, it is important to ensure that manufacturer's device interaction recommendations are followed, in addition to recent lead impedance testing to verify that the value falls within the manufacturer’s published safe range [6]. Lead impedance essentially refers to the ratio of voltage to current within the electrical circuit formed by the implanted device, and can be influenced by multiple factors. Measures of lead impedance serve as data for healthcare practitioners to recognize and diagnose related issues. Whether impedance increases or decreases and whether that change is sudden or gradual can be of great assistance in implantable electrical device diagnostics. While sudden changes can be mechanical in nature, a gradual increase in impedance can be a known phenomenon related to the slow mineralization of the device [16]. Impedance can additionally be affected by seemingly unrelated factors such as spinal cord level and time since implantation [17]. As previously alluded to, NCIEDs which are stated by the manufacturer to be conditionally compatible with other medical devices that can induce electrical current (e.g. MRI-compatible SCS) have a certain impedance range within which they are considered compatible. It has been observed that these devices frequently leave their verified impedance range, leading to unpredictable interactions [18]. At times, despite thoughtful perioperative care, NCIED-related safety events can still occur. If interference is suspected, post-operative device interrogation should be conducted and a specialist consulted to ensure that the device's position, settings, and lead impedances are appropriate [19].

## Conclusions

The open CABG surgery was safely performed on a 56-year-old male patient with two NCIEDs, namely a DBS and a recently placed SCS. It is of utmost importance for the peri- and intra-operative care teams to be aware of such implanted devices and to cooperate in their appropriate management to minimize the risk of damage to the devices and the surrounding tissue, most especially when devices are intracranial or neuraxial in location. Guideline-backed perioperative actions, including turning the devices off, placing them into safe mode, or minimizing their amplitude; identifying potential sources of current and minimizing their potential to cause harm; discussing and identifying lower risk alternatives with the surgical team; and performing device interrogation as appropriate, can ensure that patient safety and device function are maintained.
